# Hepatitis A in Spain: Evolution of hospitalization in the period 2000–2021

**DOI:** 10.1371/journal.pone.0332317

**Published:** 2025-09-24

**Authors:** Angela Domínguez, Núria Torner, Núria Soldevila, Carmen Varela, María Guerrero-Vadillo, Marina Peñuelas, Ana Avellón, Eva Borràs, Ana Martínez, Pedro Plans, Carles Pericas, Cristina Rius, Pere Godoy

**Affiliations:** 1 Departament de Medicina, Universitat de Barcelona, Barcelona, Spain; 2 CIBER Epidemiología y Salud Pública (CIBERESP), Instituto de Salud Carlos III, Madrid, Spain; 3 Centro Nacional de Epidemiología, Instituto de Salud Carlos III, Madrid, Spain; 4 Hepatitis Unit, National Centre of Microbiology, Instituto de Salud Carlos III, Madrid, Spain; 5 Agència de Salut Pública de Catalunya, Barcelona, Spain; 6 Agència de Salut Pública de Barcelona, Barcelona, Spain; 7 Institut de Recerca de l’Hospital de la Santa Creu i Sant Pau (IRB Sant Pau), Barcelona, Spain; 8 Universitat Pompeu Fabra, Barcelona, Spain; 9 Institut de Recerca Biomédica de Lleida (IRBLleida), Lleida, Spain; Pescara General Hospital, ITALY

## Abstract

**Background:**

Hepatitis A is an acute disease of the liver caused by the hepatitis A virus (HAV). Chronic liver disease, other viral hepatitis coinfections, and age over 50 years are the main host factors associated with an increased risk of complications. We investigated the evolution of hepatitis A hospitalizations and in-hospital deaths during 2000–2021 in Spain according to demographic characteristics, presence of other sexually transmitted infections, and vaccination strategy (universal or risk-group vaccination).

**Methods:**

Using data from the Spanish National Health System’s Minimum Basic Data Set, we calculated age-standardized cumulative hospitalization incidence and 95% confidence interval (CI), factors associated with hospital stay, and hospitalization deaths. Adjusted OR (aOR) values were calculated using a multivariate logistic regression model.

**Results:**

The Spanish cumulative hospitalization incidence for hepatitis A over the 22-year period was 8.84 per 1 000 000 globally and 12.54 and 5.26 per 1 000 000 for men and women, respectively (RR = 2.38; 95% CI: 2.28–2.50). Median length of stay was 4 days (range 0−85). Factors associated with hospitalization >7 days were age groups 40−59 and ≥60 years (aOR 1.58; 95% CI: 1.37–1.82 and aOR 5.09; 95% CI: 4.01–6.47, respectively), cirrhosis (aOR 6.11; 95% CI: 2.59–14.43), and presence of HIV and HBV (aOR 1.65; 95% CI: 1.15–2.38 and 2.01; 95% CI: 1.03–3.63, respectively). In-hospital deaths were associated with age ≥ 60 years (aOR 35.23; 95% CI: 11.12–111.58), hospitalization >7 days (aOR 4.37; 95% CI: 1.80–10.58), cirrhosis (aOR 8.84; 95% CI: 2.37–32.99), and HCV infection (aOR 8.66; 95% CI: 1.57–47.87). The cumulative hospitalization incidence was lower in regions implementing universal vaccination (RR 0.79; 95% CI: 0.75–0.84).

**Conclusion:**

Results of studies based on characteristics of hospitalized hepatitis A cases taking into account the existing prevention policies can be useful to have a better knowledge about its evolving epidemiology and to improve the prevention and control of the disease.

## Introduction

Hepatitis A is an acute disease of the liver caused by the hepatitis A virus (HAV) whose typical clinical manifestations include darkened urine, jaundice, pale stools, hepatomegaly, and tenderness. The major symptom’s determinant is age, as only 10%−50% of infections acquired before the age of 5 years are symptomatic, whereas 70%−95% of infected adults have symptoms. Complications with extrahepatic manifestations, which are rare, primarily include acute kidney failure, pleural or pericardial effusion, reactive arthritis, acute pancreatitis, cholecystitis, and neurological manifestations [1]. Pre-existing chronic liver disease, other viral hepatitis coinfections, and age over 50 years are the main host factors associated with an increased risk of complications [2–6]. The presence of sexually transmitted infections (STIs) before or during the disease course has been reported, and not only with human immunodeficiency virus (HIV), hepatitis B virus (HBV) and hepatitis C virus (HCV) [7–9], but also with syphilis, gonorrhoea, and *Chlamydia* infection, among others [10,11]. The proportion of these infections varies greatly depending mainly on the screening strategy implemented. Also requiring consideration is the fact that these infections are often indolent in onset [11] or are asymptomatic [10]. In male hepatitis A cases, the incidence of syphilis is more than 100 times that of the general male population and nearly 30 times that of gonorrhoea cases [12].

The case fatality rate for hepatitis A is low, ranging from 0.1%−1% depending on the studied population, but reaching as high as 10% in outbreaks [13].

Population groups predominantly affected by hepatitis A depend on the endemicity status of the country [1]. In developing countries, children are the most affected group and disease course is generally mild, whereas complications requiring hospitalization are more frequent in adolescents and adults [14,15].

Hepatitis A occurs worldwide, but major geographic differences exist related to endemicity, epidemiology patterns and anti-HAV immunoglobulin G (IgG) antibody prevalence. Different classifications have been used to determine endemicity level, although most frequently used are anti-HAV IgG antibody seroprevalence and incidence rates [16]. In high-income countries, HAV infection endemicity is low or very low; children infection and incidence are low, and the disease usually occurs only in sporadic outbreaks involving hepatitis A high-risk groups. High-risk groups include persons who travel from non-endemic countries to HAV endemic areas, family members and close contacts of infected individuals, men who have sex with men (MSM), patients with chronic liver disease, immunosuppressed patients living in areas of intermediate HAV endemicity, persons who inject drugs, children of migrants and family members of adoptees from HAV-endemic countries, among others. In recent years, a large number of person-to-person transmission outbreaks have been reported among MSM worldwide [1,16,17].

With an hepatitis A incidence rate below the threshold of 5 per 100 000 inhabitants [18], a value similar to those obtained in Portugal or Italy [19,20], Spain belongs to the group of very low endemicity countries, and this is also confirmed by the seroprevalence data. The seroprevalence study carried out in Spain in 2017–2018 shows that HAV infection prevalence was 22.94% in the 30–39 age group [21].

Although data on hepatitis A transmission mechanism are not regularly reported at national level there are specific studies on this [18]. A study carried out in Catalonia of cases reported during 2005–2015 [22] identified the main risk factors as travel to a hyperendemic country (24.6%), contact with a confirmed case (20.1%), MSM sexual contact (11.9%), raw shellfish consumption (8.8%), and exposure to raw sewage (0.1%). A study carried out in the Spanish province of Guadalajara [23] considering three periods 1991–1999, 2000–2008, and 2009–2017, found that food related transmission was very low (3.5% globally and 3%−6% in the different periods). Significant increases were evident in both the travel-related cases (18.2% globally, increasing from 8.6% in 1991–1999 to 36.4% in 2009–2017) and MSM-related cases (8% globally, increasing from 0% in 1991–1999 to 27.3% in 2009–2017).

A Spanish study of hepatitis A outbreaks in the period 2001–2018 [24] reported that most (84.8%) outbreaks involved person-to-person transmission, mainly MSM-related, and that 26.6% of outbreaks were imported.

Inactivated hepatitis A vaccines have been available since 1995 in Spain and vaccination is recommended as follows: persons with chronic liver disease or awaiting a liver transplant, persons with HIV, MSM, persons with multiple partners, sex workers, persons who inject drugs, people who work with non-human primates or with HAV in laboratories, and persons who travel to high or intermediate endemic countries [25]. However, strategy differences exist: most Spanish regions follow the risk-group vaccination strategy, but from 1999, Catalonia, Ceuta and Melilla opted for universal vaccination in addition to risk-group vaccination.

The aim of this study was to investigate the evolution of hospitalization and in-hospital death cumulative incidence in the period 2000–2021 in Spain according to demographic characteristics, the presence of other STIs before or during hepatitis A episode, and the implemented vaccination strategy.

## Methods

### Study design and population

In this retrospective study, carried out from 2000 to 2021, we analyzed data on acute hepatitis A hospital discharges contained in the Spanish National Health System’s Minimum Basic Data Set (MBDS). The MBDS is a compulsory administrative registry of clinical-administrative data of hospitalizations that collects sociodemographic, clinical, and administrative patient data from the National Health System discharge reports.

### Data collection

Minimum Basic Data Set diagnoses and procedures were coded using the International Classification of Diseases, 9th Revision, Clinical Modification (ICD-9-CM) from 2000 to 2015, and from 2016, using the International Classification of Diseases, 10th Revision (ICD-10).

Only patients with a primary diagnosis of acute hepatitis A were included (ICD-9: 0.70.0 Hepatitis A with hepatic coma and 0.70.1 Hepatitis A without hepatic coma; ICD-10: B15.0 Hepatitis A with hepatic coma and B15.9 Hepatitis A without hepatic coma).

The following relevant secondary diagnoses were also analyzed: Cirrhosis (ICD-9: 571.2, 571.5 and 571.6; ICD-10: K70.3, K74.3, K74.4, K74.5 and K74.6), HIV (ICD-9: 042; ICD-10: B20), HBV (ICD-9: 070.22, 070.23, 070.32, and 070.33; ICD-10: B18.0 and B18.1), HCV (ICD-9: 070.54; ICD-10: B18.2), syphilis (ICD-9: 091, 092, 093, 094, 095, 096, and 097; ICD-10: A51, A52, and A53), gonorrhoea (ICD-9: 098.0, 098.1, 098.2, 098.3, 098.5, 098.6, 098.7, and 098.8; ICD-10: A54.0, A54.1, A54.2, A54.4, A54.5, A54.6, A54.7, A54.8, and A54.9), and *Chlamydia* infection (ICD-9: 099.1 and 099.5; ICD-10: A55 and A56). Liver transplant (LTx) was also included as a hospital admission procedure (ICD-9: 50.5; ICD-10: 0FY00Z).

The studied variables were sex, age, region of residence, hospital admission and discharge dates, and death. Hospital stay was considered lengthy if length of stay (LOS) was > 7 days.

No exclusions were made due to missing data, as all records in the MBDS database were complete for the analyzed variables.

### Statistical analysis

Categorical variables were presented as total number and percentage, and continuous variables as median and range.

The age-standardized cumulative hospitalization incidence was calculated per 1 000 000 persons by year, sex, and vaccination strategy (universal vaccination in Catalonia, Ceuta, and Melilla or risk-group vaccination in the rest of the regions).

Cumulative hospitalization incidence was compared by calculating risk ratio (RR) and the corresponding 95% confidence interval (CI). Crude odds ratio (OR), adjusted OR (aOR), and 95% CI values were estimated using logistic regression. Adjusted OR values were calculated using the logistic regression model with backward variable selection, for a cut-off point of p < 0.2. The variables considered for the model were age, sex, LOS, cirrhosis, liver transplant, and STI. The aging index variable was also considered to adjusted the model, the aging index was defined as the number of individuals aged 65 years and over per 100 individuals under age 15, and was retrieved from annual national demographic data. Differences were considered significant when the p value was less than 0.05.

Analyses were performed using the SPSS v.29 statistical package and OpenEpi v.3.01.

### Ethics approval

According to the Spanish legislation, no ethical approval was needed for this study because it was based on analysis of the data obtained from the MBDS of the Ministry of Health of Spain. Investigators requested the hospital discharge database from the Spanish Ministry of Health by completing and application form [26]. Individual informed consent is not required for data to be included in MBDS. The data were completely anonymous, and this study used data with no identifiable information on the participants.

## Results

A total of 8841 hepatitis A hospitalizations corresponding to the primary diagnosis were recorded during the 22-year study period, 69.8% (n = 6170) men. The 20–39 age group was proportionately the largest age group (50.6%; n = 4476).

**Fig 1:**
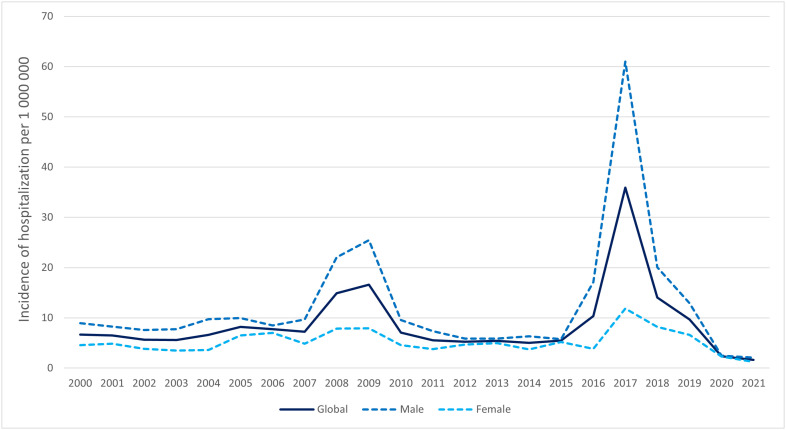
Cumulative incidence of hepatitis A hospitalization, Spain 2000–2021.

Fig 1 depicts hepatitis A cumulative hospitalization incidence per 1 000 000 over the study period, showing a slight increase in 2008 and 2009 (14.88 and 16.58 per 10^6^, respectively) and a large increase in 2017 (35.92 per 10^6^). Distribution by sex showed a higher cumulative hospitalization incidence in men, both annually and in the three years when incidence increased, i.e., 2008, 2009, and 2017. The cumulative hospitalization incidence per 1 000 000 during the entire study period was 12.54 in men and 5.26 in women (RR = 2.38; 95% CI: 2.28–2.50; p < 0.001).

Table 1 summarizes hospitalized patient characteristics by sex. Compared with the reference 20–39 age group, the other age groups had less hospitalizations in men than in women. Length of stay >7 days for men was less frequent than for women (aOR 0.84; 95% CI: 0.74–0.95). Sexually transmitted infections were more frequent in men than in women and those more frequently associated with men were HIV (aOR 6.03; 95% CI: 2.95–12.61), HBV (aOR 6.83; 95% CI: 1.63–28.61), and syphilis (aOR 4.35; 95% CI: 1.57–12.06). There were no significant differences in cirrhosis, death and LTx according to sex.

**Table 1:** Hepatitis A hospitalized patient characteristics by sex, Spain 2000–2021.

	All N = 8841	Male N = 6170	Female N = 2671	OR (95% CI)	P	aOR (95% CI)	P
Age*	29 (0-99)	30 (0-93)	26 (0-99)		**<0.001**		
**Age group**							
< 1 year	12 (0.1%)	6 (0.1%)	6 (0.2%)	**0.29 (0.09-0.90)**	**0.03**	**0.31 (0.10-0.96)**	**0.04**
1–4 years	333 (3.8%)	182 (2.9%)	151 (5.7%)	**0.35 (0.28-0.44)**	**<0.001**	**0.37 (0.29-0.46)**	**<0.001**
5–19 years	1893 (21.4%)	1040 (16.9%)	853 (31.9%)	**0.35 (0.31-0.39)**	**<0.001**	**0.36 (0.32-0.41)**	**<0.001**
20–39 years	4476 (50.6%)	3474 (56.3%)	1002 (37.5%)	Ref.		Ref.	
40–59 years	1803 (20.4%)	1301 (21.1%)	502 (18.8%)	**0.75 (0.66-0.85)**	**<0.001**	**0.72 (0.63-0.82)**	**<0.001**
≥ 60 years	324 (3.7%)	167 (2.7%)	157 (5.9%)	**0.31 (0.24-0.39)**	**<0.001**	**0.34 (0.27-0.43)**	**0.006**
LOS*	4 (0-85)	4 (0-85)	4 (0-78)		0.70		
LOS > 7 days	1507 (17.0%)	1011 (16.4%)	496 (18.6%)	**0.86 (0.76-0.97)**	**0.01**	**0.84 (0.74-0.95)**	**0.001**
LOS ≤ 7 days	7334 (83.0%)	5159 (83.6%)	2175 (81.4%)	Ref.		Ref.	
**Death**							
Yes	29 (0.3%)	13 (0.2%)	16 (0.6%)	**0.35 (0.17-0.73)**	**0.005**	0.56 (0.25-1.23)	0.15
No	8812 (99.7%)	6157 (99.8%)	2655 (99.4%)	Ref.		Ref.	
**LTx**							
Yes	16 (0.2%)	10 (0.2%)	6 (0.2%)	0.72 (0.26-1.99)	0.53		
No	8825 (99.8%)	6160 (99.8%)	2665 (99.8%)	Ref.			
**Cirrhosis**							
Yes	27 (0.3%)	19 (0.3%)	8 (0.3%)	1.03 (0.45-2.35)	0.95		
No	8814 (99.7%)	6151 (99.7%)	2663 (99.7%)	Ref.			
**Any STI**							
Yes	312 (3.5%)	291 (4.7%)	21 (0.8%)	**6.25 (4.00-9.75)**	**<0.001**		
No	8529 (96.5%)	5879 (95.3%)	2650 (99.2%)	Ref.			
**HIV**							
Yes	163 (1.8%)	155 (2.5%)	8 (0.3%)	**8.58 (4.21-17.48)**	**<0.001**	**6.03 (2.95-12.61)**	**<0.001**
No	8678 (98.2%)	6015 (97.5%)	2663 (99.7%)	Ref.		Ref.	
**HBV**							
Yes	43 (0.5%)	41 (0.7%)	2 (0.1%)	**8.93 (2.16-36.93)**	**0.003**	**6.83 (1.63-28.61)**	**0.01**
No	8798 (99.5%)	6129 (99.3%)	2669 (99.9%)	Ref.		Ref.	
**HCV**							
Yes	67 (0.8%)	58 (0.9%)	9 (0.3%)	**2.81 (1.39-5.67)**	**0.004**	1.85 (0.90-3.83)	0.10
No	8774 (99.2%)	6112 (99.1%)	2662 (99.7%)	Ref.		Ref.	
**Syphilis**							
Yes	65 (0.7%)	61 (1.0%)	4 (0.1%)	**6.66 (2.42-18.33)**	**<0.001**	**4.35 (1.57-12.48**06	**0.005**
No	8776 (99.3%)	6109 (99.0%)	2667 (99.9%)	Ref.		Ref.	
**Gonorrhoea**							
Yes	4 (0.04%)	3 (0.05%)	1 (0.04%)	1.30 (0.14-12.49)	0.82		
No	8837 (99.96%)	6167 (99.95%)	2670 (99.96%)	Ref.			
***Chlamydia*** infection							
Yes	3 (0.03%)	3 (0.05%)	0 (0.0%)	–	0.56		
No	8838 (99.97%)	6167 (99.95%)	2671 (100%)	Ref.			

* Values expressed as median (range). HBV: hepatitis B virus; HCV: hepatitis C virus; HIV: human immunodeficiency virus; LOS: length of stay; LTx: liver transplant; STI: sexually transmitted infection.

Table 2 summarizes factors associated with LOS > 7 days. The median was 4 days (range 0–85) for all age groups and 3 days (range 0–85) for <15 years. Factors associated with LOS > 7 days were age groups 40–59 years (aOR 1.58; 95% CI: 1.37–1.82) and ≥60 years (aOR 5.09; 95% CI: 4.01–6.47), cirrhosis (aOR 6.11; 95% CI: 2.59–14.43), HIV infection (aOR 1.65; 95% CI: 1.15–2.38) and HBV infection (2.01; 95% CI; 1.03–3.63). Age 1–4 years, 5–19 years, and being male were negatively associated with a high LOS value.

**Table 2:** Factors associated with LOS in hepatitis A hospitalized cases, Spain 2000–2021.

	LOS > 7 days N = 1507	LOS ≤ 7 days N = 7334	OR (95% CI)	P	aOR (95% CI)	P
Age*	34 (0-96)	29 (0-99)		**<0.001**		
**Age group**						
<1 year	2 (0.1%)	10 (0.1%)	1.03 (0.23-4.72)	0.97	0.96 (0.21-4.50)	0.95
1–34 years	26 (1.7%)	307 (4.2%)	**0.44 (0.29-0.66)**	**<0.001**	**0.40 (0.27-0.60)**	**<0.001**
5–19 years	190 (12.6%)	1703 (23.2%)	**0.58 (0.49-0.68)**	**<0.001**	**0.56 (0.47-0.66)**	**<0.001**
20–39 years	727 (48.2%)	3749 (51.1%)	Ref.		Ref.	
40–59 years	401 (26.6%)	1402 (19.1%)	**1.48 (1.29-1.69)**	**<0.001**	**1.58 (1.37-1.6782**	**<0.001**
≥60 years	161 (10.7%)	163 (2.2%)	**5.09 (4.04-6.42)**	**<0.001**	**5.09 (4.01-6.47)**	**<0.001**
**Sex**						
Male	1011 (67.1%)	5159 (70.3%)	**0.86 (0.76-0.97)**	**0.01**	**0.83 (0.74-0.95)**	**0.005**
Female	496 (32.9%)	2178 (29.7%)	Ref.		Ref.	
**LTx**						
Yes	16 (1.1%)	0 (0.0%)	**–**	**<0.001**		
No	1491 (98.9%)	7334 (100%)	Ref.			
**Cirrhosis**						
Yes	19 (1.3%)	8 (0.1%)	**11.69 (5.11-26.76)**	**<0.001**	**6.11 (2.59-14.43)**	**<0.001**
No	1488 (98.7%)	7326 (99.9%)	Ref.			
**Any STI**						
Yes	72 (4.8%)	240 (3.3%)	**1.48 (1.13-1.94)**	**0.004**		
No	1435 (95.2%)	7094 (96.7%)	Ref.			
**HIV**						
Yes	41 (2.7%)	122 (1.7%)	**1.65 (1.16-2.37)**	**0.006**	**1.65 (1.15-2.38)**	**0.01**
No	1466 (97.3%)	7212 (98.3%)	Ref.		Ref.	
**HBV**						
Yes	14 (0.9%)	29 (0.4%)	**2.36 (1.24-4.48)**	**0.01**	**2.01 (1.03-3.63)**	**0.04**
No	1493 (99.1%)	7305 (99.6%)	Ref.		Ref.	
**HCV**						
Yes	18 (1.2%)	49 (0.7%)	**1.80 (1.04-3.09)**	**0.03**		
No	1489 (98.8%)	7285 (99.3%)	Ref.			
**Syphilis**						
Yes	10 (0.7%)	55 (0.7%)	0.88 (0.45-1.74)	0.72		
No	1497 (99.3%)	7279 (99.3%)	Ref.			
**Gonorrhoea**						
Yes	1 (0.1%)	3 (0.04%)	1.62 (0.17-15.61)	0.67		
No	1506 (99.9%)	7331 (99.96%)	Ref.			
***Chlamydia* infection**						
Yes	1 (0.1%)	2 (0.03%)	2.43 (0.22-26.86)	0.47		
No	1506 (99.9%)	7332 (99.97%)	Ref.			

* Values expressed as median (range). HBV: hepatitis B virus; HCV: hepatitis C virus; HIV: human immunodeficiency virus; LOS: length of stay; LTx: liver transplant; STI: sexually transmitted infection.

Table 3 reports that the factors associated with hospital deaths were age ≥ 60 years (aOR 35.23; 95% CI: 11.12–111.58), LOS > 7 days (aOR 4.37; 95% CI: 1.80–10.58), cirrhosis (aOR 8.84; 95% CI: 2.37–32.99), and HCV (aOR 8.66; 95% CI: 1.57–47.87).

**Table 3:** Factors associated with death during hepatitis A hospitalization, Spain 2000–2021.

	Death N = 29	No death N = 8812	OR (95% CI)	P	aOR (95% CI)	P
**Age**	67 (18-89)	29 (0-99)		**<0.001**		
Age group*						
<1 year	0 (0%)	12 (0.1%)	–		–	
1–4 years	0 (0%)	333 (3.8%)	–		–	
5–19 years	1 (3.4%)	1892 (21.5%)	0.59 (0.07-5.29)	0.64	0.63 (0.07-5.76)	0.68
20–39 years	4 (13.8%)	4472 (50.7%)	Ref.		Ref.	
40–59 years	4 (13.8%)	1799 (20.4%)	2.48 (0.62-9.95)	0.20	2.08 (0.51-8.55)	0.31
≥60 years	20 (69.0%)	304 (3.4%)	**73.55 (24.98-216.54)**	**<0.001**	**35.23 (11.12-111.58)**	**<0.001**
**Sex**						
Male	13 (44.8%)	6157 (69.9%)	**0.35 (0.17-0.73)**	**0.005**	0.46 (0.21-1.04)	0.06
Female	16 (55.2%)	2655 (30.1%)	Ref.		Ref.	
LOS*	12 (2-64)	4 (0-85)		**<0.001**		
LOS > 7 days	21 (72.4%)	1486 (16.9%)	**12.94 (5.72-29.27)**	**<0.001**	**4.37 (1.80-10.58)**	**0.001**
LOS ≤ 7 days	8 (27.6%)	7326 (83.1%)	Ref.		Ref.	
**LTx**						
Yes	0 (0%)	16 (0.2%)	–	1.00		
No	29 (100%)	8796 (99.8%)	Ref.			
**Cirrhosis**						
Yes	4 (13.8%)	23 (0.3%)	**61.14 (19.71-189.65)**	**<0.001**	**8.84 (2.37-32.99)**	**0.001**
No	25 (86.2%)	8789 (99.7%)	Ref.		Ref.	
**Any STI**						
Yes	2 (6.9%)	310 (3.5%)	2.03 (0.48-8.58)	0.34		
No	27 (93.1%)	8502 (96.5%)	Ref.			
**HIV**						
Yes	1 (3.4%)	162 (1.8%)	0.52 (0.07-3.88)	0.53		
No	28 (96.6%)	8650 (98.2%)	Ref.			
**HBV**						
Yes	1 (3.4%)	42 (0.5%)	7.46 (0.99-56.08)	0.05		
No	28 (96.6%)	8770 (99.5%)	Ref.			
**HCV**						
Yes	2 (6.9%)	65 (0.7%)	**9.97 (2.32-42.79)**	**0.002**	**8.66 (1.57-47.87)**	**0.01**
No	27 (93.1%)	8747 (99.3%)	Ref.		Ref.	
**Syphilis**						
Yes	0 (0%)	65 (0.7%)	–	1.00		
No	29 (100%)	8747 (99.3%)	Ref.			
**Gonorrhoea**						
Yes	0 (0%)	4 (0.04%)	–	1.00		
No	29 (100%)	8808 (99.96%)	Ref.			
***Chlamydia* infection**						
Yes	0 (0%)	3 (0.03%)	–	1.00		
No	29 (100%)	8809 (99.97%)	Ref.			

* Values expressed as median (range). HBV: hepatitis B virus; HCV: hepatitis C virus; HIV: human immunodeficiency virus; LOS: length of stay; LTx: liver transplant; STI: sexually transmitted infection.

Table 4 reports cumulative hospitalization incidence according to vaccination strategy by year. Incidence was significantly higher in regions with universal vaccination than in those with vaccination of risk groups in 2000, 2002, 2003 and 2004, and significantly lower in 2001, 2007–2009, 2013 and 2016–2020.

**Table 4:** Age-standardized hepatitis A cumulative hospitalization incidence (per 1 000 000) by year and by vaccination strategy of the region, Spain 2000–2021.

	Regions with universal vaccination strategy	Regions with risk-group vaccination strategy	RR (95% CI)	P
2000	12.16	5.71	**2.13 (1.83-2.50)**	**<0.001**
2001	5.33	6.74	**0.79 (0.66-0.95)**	**0.01**
2002	9.31	4.99	**1.87 (1.59-2.22)**	**<0.001**
2003	8.63	5.04	**1.70 (1.44-2.202**	**<0.001**
2004	7.94	6.37	**1.25 (1.06-1.46)**	**0.01**
2005	7.75	8.29	0.94 (0.80-1.09)	0.39
2006	7.02	7.90	0.89 (0.76-1.04)	0.14
2007	5.46	7.58	**0.72 (0.61-0.85)**	**<0.001**
2008	7.55	16.33	**0.46 (0.41-0.52)**	**<0.001**
2009	11.95	17.50	**0.68 (0.61-0.76)**	**<0.001**
2010	7.11	7.02	1.01 (0.87-1.18)	0.88
2011	4.77	5.68	0.84 (0.70-1.01)	0.06
2012	5.96	5.13	1.16 (0.98-1.38)	0.09
2013	4.47	5.60	**0.80 (0.67-0.96)**	**0.02**
2014	5.40	4.93	1.10 (0.92-1.32)	0.32
2015	5.12	5.53	0.93 (0.78-1.10)	0.39
2016	5.14	11.39	**0.45 (0.39-0.52)**	**<0.001**
2017	19.12	39.30	**0.49 (0.45-0.53)**	**<0.001**
2018	9.24	15.00	**0.62 (0.55-0.69)**	**<0.001**
2019	7.86	10.08	**0.78 (0.68-0.89)**	**0.01**
2020	1.77	2.51	**0.71 (0.53-0.93)**	**0.02**
2021	2.02	1.57	1.28 (0.95-1.74)	0.10

Finally, Table 5 reports cumulative hospitalization incidence by age, sex, LOS > 7 days, and deaths according to vaccination strategy. Cumulative incidence was lower in regions with universal vaccination, both overall (RR 0.79; 95% CI: 0.77–0.82) and separately for men (RR 0.76; 95% CI: 0.73–0.78) and women (RR 0.88; 95% CI: 0.83–0.93). In regions with universal vaccination, cumulative incidence was also lower for cases with LOS > 7 days (RR 0.57; 95% CI: 0.52–0.62).

**Table 5:** Age-standardized hepatitis A cumulative hospitalization incidence (per 1 000 000) by vaccination strategy, Spain 2000–2021.

	Regions with universal vaccination strategy N (Cumulative incidence)	Regions with risk-group vaccination strategy N (Cumulative incidence)	RR (95% CI)	P
**Total hospitalization**	1187 (7.26)	7654 (9.15)	**0.79 (0.77-0.82)**	**<0.001**
**Age group**				
<1 year	1 (0.63)	11 (1.54)	0.41 (0.15-1.10)	0.07
1–4 years	69 (10.33)	264 (8.37)	1.23 (0.98-1.43)	0.06
5–19 years	254 (10.45)	1639 (13.01)	**0.80 (0.75-0.86)**	**<0.001**
20–39 years	544 (11.35)	3932 (16.23)	**0.70 (0.67-0.73)**	**<0.001**
40–59 years	259 (5.63)	1544 (6.51)	**0.86 (0.81-0.92)**	**0.001**
≥60 years	60 (1.63)	264 (1.37)	1.19 (0.97-1.39)	0.06
**Sex**				
Male	796 (9.87)	5374 (13.06)	**0.76 (0.73-0.78)**	**<0.001**
Female	391 (4.73)	2280 (5.36)	**0.88 (0.83-0.93)**	**0.001**
**LOS > 7 days**	151 (0.92)	1356 (1.62)	**0.57 (0.52-0.62)**	**<0.001**
**Hospital deaths**	8 (0.05)	21 (0.03)	1.95 (0.95-3.25)	0.08

LOS: length of stay.

## Discussion

While the cumulative hospitalization incidence for hepatitis A was higher in men than in women during 2000–2019, no differences were observed for 2020 and 2021, i.e., years with very low hospitalization rates due to the COVID-19 pandemic. The decrease in these years, in both regions with a universal and with a risk-group vaccination strategy, is corroborated by studies carried out in Spain and Denmark, where hospital admission for non-COVID-19 medical conditions was at very low levels [27,28]. Reported hepatitis A cases also decreased in those years [18].

Over the entire 22-year study period, the cumulative hospitalization incidence was higher in men (12.54 per 1 000 000) than in women (5.26 per 1 000 000), a finding that has also been reported for other authors [29].

In the present study the lower hospitalization proportion corresponds to children under 5 (3.9% of all hospitalizations) and the highest percentage to the 20–39 age group (50.6% of all hospitalizations). Similar to our results, in the study by Thompson et al. [30] carried out in Australia between 2000–2014, the highest percentage of hospitalization in the population that benefited from a vaccination programme starting in 2005 corresponded to the 20–49 age group.

Coinciding with our results, in a study of three USA states carried out in 2016–2019, Hofmeister et al. [31] found that the highest proportion of hospitalized patients were from the 20–39 age group. Our results also agree with the finding that hepatitis A hospitalization was highest in the 18–39 age group in most European countries [29].

Hospitalization is unnecessary in the absence of severe disease or acute liver failure, as therapy is aimed at maintaining adequate nutritional balance and replacing fluids lost due to vomiting and diarrhoea [32]. Rodriguez Tajes et al. reported in a case series, where the median age was 33 years (similar to that obtained in our study), that 50% of hospitalized patients had severe hepatitis (defined as impaired liver function with jaundice and coagulopathy, according to the European Association for the Study of the Liver), while the remaining cases were hospitalized due to gastrointestinal symptoms or dehydration [33].

A study conducted in an academic hospital in Barcelona analyzing patients attending the emergency department during the period 2014–2018 found that among viral hepatitis cases, hepatitis A had the lowest proportion of hospital admissions [34]. Other authors [31,35] have stated that being MSM, drug users or experiencing homelessness are factors associated with increased risk of hospitalization. Furthermore, it has been pointed out that individuals with limited or difficult access to primary care are more likely to seek medical attendance in emergency departments [36,37].

Inpatient hospital mortality in our study was 0.33%, and deaths were associated with age ≥ 60 years, males, and LOS > 7 days. In the USA, older age (≥55 years during 1998–2020 and >60 years during 2002–2013) [38,39] was reported to be a risk factor for inpatient mortality, corroborating other studies that likewise underscore the increased acute hepatitis A mortality risk associated with age [40,41]. For European countries, Severi et al. [29] found that the highest case fatality rate was observed in people aged ≥50 years, and Andani et al. [42] reported that 69% of deaths occurred in people ≥60 years.

In the present study median LOS was 4 days (range 0–85) and LOS was ≥ 7 days in 17% of cases. In the Spanish study by García-Ferreira et al. [43] in 2016 in Andalusia, a median LOS of 3 days for hepatitis A hospitalizations was observed, while Martínez-Lozano et al. [44] in Madrid during the period 2005–2017 reported a LOS of 5.02 days. For the USA, the median LOS was 4 days [38], and for Europe, proportion of LOS > 7 days in hospitalized patients ranged from 11% to 58% [29]. In contrast to the finding of no difference between Taiwanese males and females by Chen et al. [45], we observed a lower frequency of LOS > 7 days in men. In the study by Ofori-Asenso et al. carried out in Australia [46] female gender was also a factor independently associated to prolonged hospitalization for admission episodes of all diseases. Length of stay was lower for the 1–4 and 5–19 age groups and higher for the 40–59 and ≥60 age groups.

For children and adolescents <15 years hospitalized for hepatitis A median LOS was 3 days (range 0–85) in the present study. Papaevangelou et al. [47] and Braccio et al. [48] reported a median LOS of 5 days for Greece and a median LOS of 1 day for the UK, respectively.

In our study, 3.5% of hepatitis A hospitalized cases had an STI. This proportion that corresponds to those hospitalized in general population, is lower than that reported by some authors in small series of outbreak related cases [9,10,33,49]. Length of stay of hospitalized cases with STI was longer than for patients without an STI. HBV, HCV, HIV, and syphilis were more frequent in men than women. Length of stay was similar for patients with syphilis, gonorrhoea or *Chlamydia* infection, but was higher for patients with HIV, HBV, or HCV. Patients with HCV had a higher risk of inpatient death than the remaining patients with STIs.

Hepatitis A vaccination of HIV positive patients has been recommended in Spain since 2018 [25] and although the incidence of hepatitis A in people living with HIV (PLWH) is low, some available data [50] show that those cases correspond to unvaccinated subjects. In Portugal, around 70 cases in an important outbreak during October 2023-April 2024 were associated with sexual transmission in MSM and 20% of them were HIV positive [51]. Strategies to reinforce HAV vaccination are needed for HIV-infected people. Both MSM living with HIV and HIV-uninfected receiving HIV pre-exposure prophylaxis (PrEP) are vulnerable to HAV infection, and so may be a potential source of a more widespread HAV epidemic [52]. It would be important to follow recommendations for hepatitis A vaccination in people living with HIV. In Spain, since 2018 hepatitis A vaccination was recommended for this population group [53]. However, it is recognized that the vaccine may not provide long-term protection, and individuals should undergo a post-vaccination serologic test after completing the hepatitis A vaccine series. In Spain it is recommended the post-vaccination serologic test two or more months after completing vaccination series and an additional dose of vaccine if result of serologic test is negative [53]. In the United States this serologic-vaccination test is recommended one month or more after completing vaccination and the administration of a new dose if the result is negative; additionally, it is indicated that regardless of results of serologic test, these individuals might need to receive immune globulin administration after a high-risk exposure, such as sexual or household contact [54]. For vaccinated individuals who remain at continued risk of hepatitis A, a booster dose every 10 years has also been proposed [55], although supporting evidence for this recommendation is currently lacking [56]. Some authors [57] have recently proposed that post-vaccination antibody assessment should be limited to patients receiving immunosuppressive combination therapy or those who are immunocompromised, but, the number of subjects included in their study was small, and this aspect should be more widely studied. A level of >70% immunity in MSM has been proposed to prevent sustained hepatitis A transmission [58,59]. However, a French study [60] suggests that a 70% threshold may be insufficient to prevent outbreaks and that age structure and behavioural and socio-demographic characteristics need to also be taken into account.

In a study by Marciano et al. [61] carried out in Argentina in 2017–2018, 23% and 26% of diagnosed hepatitis A cases (35% requiring hospitalization) tested positive for HIV and syphilis, respectively. In France, in a study carried out in 2017, the frequency of hepatitis A in HIV-infected patients was 0.86%, increasing to 3% in MSM [7].

The annual cumulative hepatitis A hospitalization incidence was only significantly greater in regions with universal vaccination in years 2000, 2002, 2003, and 2004, i.e., when few years had elapsed since universal vaccination was launched. No differences were found until outbreaks affecting mainly MSM occurred in 2007–2009 and 2016–2018, years with a lower cumulative hospitalization incidence in regions with universal vaccination.

Hospitalization incidence was lower in the Spanish regions with universal vaccination. The fact that LOS > 7 days was also less frequent in regions with universal vaccination would indicate mitigation of the hepatitis A hospitalization burden, as a benefit of universal vaccination. However, the influence of outbreaks affecting MSM cannot be underestimated and possible specific high-risk vaccination campaigns in addition to universal vaccination strategy might have influenced.

Since outbreaks affecting several countries still occur despite the availability of safe and effective vaccines [9,49,62,63], resources need to be designated to both vaccinating and raising awareness among people at high risk [35,43].

In 2016, the 194 member states of the World Health Organization (WHO) committed to eliminating viral hepatitis as a public health threat by 2030. Because HAV endemicity is low in most developed countries, substantial proportions of their populations are vulnerable. However, vaccination recommendations are heterogeneous, and there is a need to improve monitoring of hospitalizations and vaccination coverage in specific risk groups in order to achieve WHO’s goal of eliminating viral hepatitis by 2030 [64–66].

This study has some limitations. Firstly, due to the retrospective nature of the discharge records, certain data potentially related with the risk of exposure to hepatitis A and STIs were not available, such as country of origin, socioeconomic status, sexual behaviours, travel history or vaccination status. Integration of vaccination information in hospitalizations data set would allow for a more robust evaluation of the impact of the vaccination programme in the future. Vaccination coverage is not available at national level because the vaccine is only recommended to risk groups. Another limitation is that we did not have clinical data and, therefore, it was not possible to know how many STIs had clinical manifestations and how many were asymptomatic. A final limitation is that, since the hospital discharge records only report inpatient mortality, the real mortality burden due to deaths occurring after discharge is underestimated.

The main strength of our study is the universal data coverage: the fact that all discharges of the National Health System (which attend 96% of the population [67]) are recorded means a very low risk of underestimating the hepatitis A hospitalization burden. In addition, our study only included discharges with hepatitis A as the main diagnosis, which would guarantee having avoided overestimation of the hepatitis A hospitalization burden.

In conclusion, presence of other agents that cause STIs lead to a greater hospitalization risk and a longer hospital stay, and in the case of HCV, a greater in-hospital risk. Results of studies based on characteristics of hospitalized hepatitis A cases taking into account the existing prevention policies can be useful to have a better knowledge about its evolving epidemiology and to improve the prevention and control of the disease. Even though hepatitis A immunization policies may be the responsibility of national or regional public health authorities, it would be useful to carry out cost-effectiveness studies that would bolster the response to cross-border threats. Irrespective of whether the vaccination strategy is universal or focused on high-risk groups, efforts to proactively vaccinate and raise awareness among groups at high risk of hepatitis A and other STIs should be improved.
